# Plant traits and associated ecological data from global change experiments and climate gradients in Norway

**DOI:** 10.1038/s41597-025-05509-4

**Published:** 2025-08-25

**Authors:** Vigdis Vandvik, Aud H. Halbritter, Marc Macias-Fauria, Brian S. Maitner, Sean T. Michaletz, Richard J. Telford, Nicole Bison, Julia Chacon-Labella, Sehoya Cotner, Dagmar Egelkraut, Josef Garen, Joseph Gaudard, Sonya R. Geange, Maria A. Rosati, Emil A. S. Andersen, Sam J. Ahler, Joe Atkinson, Marta Baumane, Pia M. Bradler, Hilary Rose Dawson, Julia Eckberg, Alexander D. Elsy, Joshua Erkelenz, Susan E. Eshelman, Coskun Guclu, Rebekka Gullvåg, Ragnhild Gya, Sorrel Hartford, Meghan T. Hayden, Mukhlish J. M. Holle, Alyssa T. Kullberg, Kai Lepley, Marta Correia, Cora E. Löwenstein, Celesté Maré, Dickson Mauki, Jocelyn Navarro, Barryette Oberholzer, Bernard Olivier, Alyssa N. Olson, Courtenay A. Ray, Jonathan von Oppen, Tom Vorstenbosch, Jonathan A. Wang, Brian J. Enquist

**Affiliations:** 1 Department of Biological Sciences, University of Bergen, Bergen, Norway; 2 Bjerknes Centre for Climate Research, University of Bergen, Bergen, Norway; 3 Scott Polar Research Institute, University of Cambridge, Cambridge, USA; 4 Department of Ecology and Evolutionary Biology, University of Arizona, Tucson, USA; 5 Department of Botany, University of British Columbia, Vancouver, Canada; 6 Biodiversity Research Centre, University of British Columbia, Vancouver, Canada; 7 Department of Botany and Biodiversity Research Centre, University of British Columbia, Vancouver, Canada; 8 Facultad de Ciencias, Departamento de Biología, Universidad Autonoma de Madrid, Madrid, Spain; 9 Climate Impacts Research Centre, Department of Ecology and Environmental Science, Umeå University, Umeå, Sweden; 10 Department of Ecology and Evolutionary Biology, University of Colorado - Boulder, Boulder, USA; 11 Institute of Arctic and Alpine Research, University of Colorado - Boulder, Boulder, USA; 12 Center for Ecological Dynamics in a Novel Biosphere (ECONOVO), Department of Biology, Aarhus University, Aarhus, Denmark; 13 Evolution & Ecology Research Centre, UNSW Sydney, Kensington, Australia; 14 Department of Biology, University of Copenhagen, Copenhagen, Denmark; 15 Department of Biology, Aarhus University, Aarhus, Denmark; 16 Institute of Ecology, Leuphana University of Lüneburg, Lüneburg, Germany; 17 Institute of General Ecology and Environmental Protection, TUD Dresden University of Technology, Tharandt, Germany; 18 Institute of Ecology and Evolution, University of Oregon, Eugene, USA; 19 Research School of Biology, Australian National University, Canberra, Australia; 20 Department of Ecology and Evolutionary Biology, University of Michigan, Ann Arbor, USA; 21 Department of Environmental Systems Science, ETH Zürich, Zurich, Switzerland; 22 Department of Botany and Zoology, Masaryk University, Brno, Czech Republic; 23 Institute of Ecology and Evolution, School of Biological Sciences, The Univerity of Edinburgh, Ashworth Laboratories, Edinburgh, Scotland; 24 Department of Biological Sciences, Faculty of Science, The University of Hong Kong, Hong Kong, Hong Kong; 25 Department of Ecology and Environmental Science, Umeå University, Umeå, Sweden; 26 Department of Ecology and Evolutionary Biology, University of Colorado, Boulder, USA; 27 Faculty of Biology, Gadjah Mada University, DI Yogyakarta, Indonesia; 28 Department of Biology, University of Miami, Coral Gables, USA; 29 School of Geography, Development and Environment, University of Arizona, Tucson, USA; 30 Centre for Functional Ecology, University of Coimbra, Coimbra, Portugal; 31 Institute of Forest Ecology, Department of Forest -and Soil Sciences, University of Natural Resources and Life Sciences, Vienna, Austria; 32 Section for Ecoinformatics and Biodiversity, Department of Biology, Aarhus University, Aarhus, Denmark; 33 Senckenberg Biodiversity and Climate research center-Frankfurt, Frankfurt, Germany; 34 Department of Forest Ecology and Management, SLU, Umeå, Sweden; 35 Department of Biology Teaching and Learning, University of Minnesota, Minneapolis, USA; 36 Department of Botany, University of Wyoming, Laramie, USA; 37 Department of Environmental Sciences, University of Basel, Basel, Switzerland; 38 Department of Botany and Biodiversity Research, University of Vienna, Vienna, Austria; 39 School of Biological Sciences, University of Utah, Salt Lake City, USA; 40 The Santa Fe Institute, Santa Fe, USA

**Keywords:** Biodiversity, Climate-change ecology, Community ecology, Ecophysiology, Ecosystem ecology

## Abstract

Plant functional trait-based approaches are powerful tools to assess the consequences of global environmental changes for plant ecophysiology, population and community ecology, ecosystem functioning, and landscape ecology. Here, we present data capturing these ecological dimensions from grazing, nitrogen addition, and warming experiments conducted along a 821 m a.s.l. elevation gradient and from a climate warming experiment conducted across a 3,200 mm precipitation gradient in boreal and alpine grasslands in Vestland County, western Norway. From these systems we collected 28,762 plant and leaf functional trait measurements from 76 vascular plant species, 88 leaf assimilation-temperature responses, 577 leaf handheld hyperspectral readings, 2.26 billion leaf temperature measurements, 3,696 ecosystem CO_2_ flux measurements, and 10.69 ha of multispectral (10-band) and RGB cm-resolution imagery from 4,648 individual images obtained from airborne sensors. These data augment existing longer-term data on local climate, soils, plant populations, plant community composition, and ecosystem functioning from within the same experiments and study systems and from similar systems in other mountain regions globally.

## Background & Summary

Understanding how species, communities, and ecosystems will respond to accelerating global changes, and how these ecosystem changes will feed back to the global climate, are urgent priorities for science and society. Functional traits, defined as measurable attributes that influence individual fitness and performance, are powerful predictors of organismal and ecosystem performance and responses across environmental gradients^1^. Because plants make up the vast majority of the global terrestrial biomass^2^, their traits and performance are also key to understand and forecast terrestrial ecosystem functioning^3^. Plant trait-based approaches are now central to global change science, informing our understanding of consequences of global environmental changes for plant ecophysiology^4–6^, population and community ecology^7^, ecosystem functioning^3^, and landscape ecology^8,9^, and of the consequences of such vegetation changes for society.

Mountain ecosystems provide critically important contributions to people, including provisioning services such as water, forage, timber, food, grazing resources for wild and domestic animals and regulating services such as carbon sequestration and natural hazard protection^10–13^. High-elevation ecosystems also harbour unique biodiversity^14^ and are expected to become increasingly important as target areas for nature conservation in the face of climate change^15^. The physiological and ecological performance of cold-climate organisms are often temperature-limited, as are the rates of ecosystem processes in cold climates^16^, suggesting that mountain biota and ecosystems may respond more rapidly and intensely to warming than the global average^17–19^. Mountains therefore offer a natural laboratory to study the impacts of climate change on biodiversity, ecosystem functioning, and nature’s contributions to people.

High-elevation ecosystems are also impacted by global change drivers beyond rising temperatures, including changes in precipitation and snow, land-use, pollution, and invasive alien species, often with complex interactions between the different drivers^20–22^. To understand and predict the future of mountain ecosystems we thus need to assess the unique and combined effects of such co-occurring global change drivers on mountain biodiversity and ecosystems. This requires integrated approaches that combine experiments and gradient studies of multiple global change drivers across diverse mountain contexts^23–25^.

**Fig. 1:**
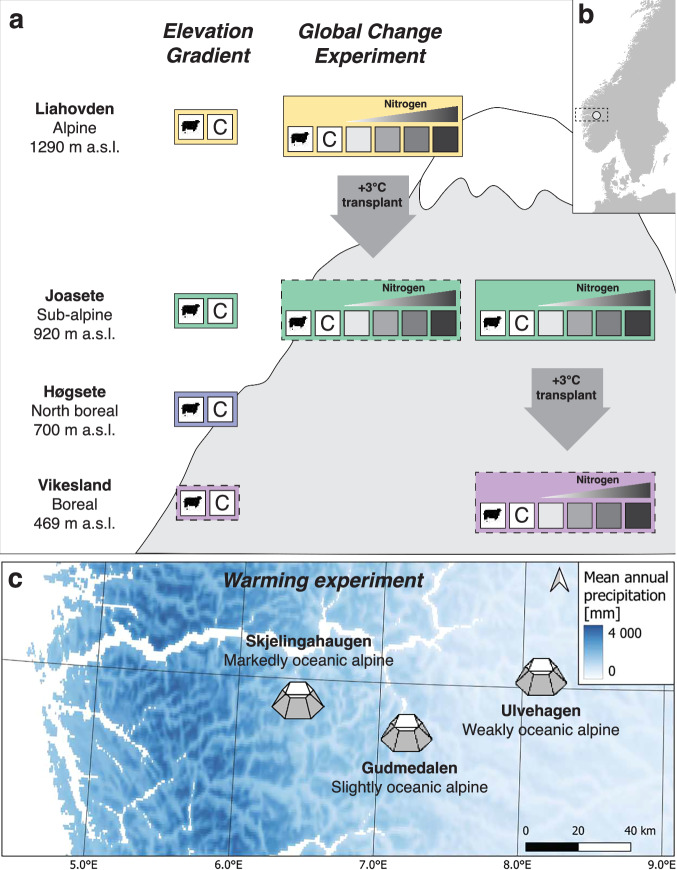
Map of sites and conceptual diagram of the experimental and study designs for the three study systems sampled for plant functional traits and associated data in the PFTC6 field campaign. (**a**) Location and study design of the Elevation Gradient and experimental design of the Global Change Experiment along a mountainside in Aurland, Vestland County, Norway. Colours (yellow, green, blue, purple) indicate the four sites. The Elevation gradient study system includes a natural grazing (sheep) treatment a fenced ungrazed control (C) and at each of the four sites. The Global Change Experiment study system includes these grazing treatments and four nitrogen addition treatments inside the ungrazed fenced area (5, 10, 50 and 150 kg N ha^−1^ yr^−1^; greyscale) in combination with a warming treatment (box transplant arrow) whereby whole-community turfs of all grazing and nitrogen treatments from the colder sites (alpine Liahovden or sub-alpine Joasete) are transplanted a 3°C ‘step’ warmer along the elevation gradient (to the sub-alpine Joasete and boreal Vikesland, respectively). On the figure, ambient climate treatments are indicated by a solid outline, warmed treatments by a dashed outline. See text for details of the hierarchical block design and replication. Note that the Global Change Experiment is based on a field experiment established in the ThreeD project, and the Elevation Gradient combines sites and grazing exclosure treatments from the ThreeD and Vestland Climate Grid projects, so that these study systems use the same plots for the ambient climate fenced control and the natural grazing treatments in the alpine Liahovden and sub-alpine Joasete sites (see Table 2 and text below). m a.s.l. = metres above sea level. (**b**) Map of southern Scandinavia with the location of the study region (dashed rectangle) and the mountainside harbouring the Elevation Gradient and Global Change Experiment (small circle) indicated. (**c**) Map of the study region with the three sites of the Warming Experiment study system indicated by Hexagonal Open Top Chambers. The Warming Experiment is based on a field experiment established in the INCLINE project (see Table 2 and text below), with five blocks of paired adjacent warmed and ambient climate plots at each site. See text for details of the hierarchical block design and replication. Bluescale reflects mean annual precipitation calculated from daily means from 2009–2019, data provided by the Norwegian Meteorological Institute (www.met.no). Note that the Elevation Gradient and Global Change Experiment are located along a mountinside just north of the Gudmedalen Warming Experiment site.

Here, we report on a plant trait-based assessment of how multiple global change drivers interactively impact mountain plants, vegetation, and ecosystems. Specifically, we combine plant and leaf functional traits with data on vegetation and ecosystem functioning within both observational and experimental settings to assess how three global change drivers, climate warming, nitrogen deposition, and grazing affect plants and ecosystems across spatial scales and organizational levels in semi-natural calcareous grasslands in Vestland County, Norway (Fig. 1a). This work makes use of established gradients and experiments and integrates with existing environmental and vegetation data from three long-term research projects: (i) the *Vestland Climate Grid; (ii)* the *ThreeD* Global Change Experiment, and (iii) the *INCLINE* Climate Warming Experiment. The Vestland Climate Grid is a grid of twelve grassland sites across broad-scale temperature and precipitation gradients across the study region, established in 2009^26–28^. The *ThreeD* Global Change Experiment combines climate warming simulated via downslope whole-community turf transplantation with nitrogen addition and grazing treatments along an elevation gradient spanning two Vestland Climate Grid sites, established in 2019. The *INCLINE* Climate Warming experiment combines Open Top Chamber (OTC) warming with novel species interaction treatments across four alpine Vestland Climate Grid sites differing by ca. 2,300 mm in annual precipitation, and was established in 2018^29^.

We made use of these existing field sites and experiments to establish three study systems: First, to study the effects of temperature in both grazed and ungrazed vegetation we combined Vestland Climate Grid and ThreeD sites to obtain four sites in Aurland, Vestland County, Norway, differing in elevation by 821 m a.s.l., each with fenced grazing exclosures and unfenced control plots (referred to as the ***Elevation Gradient***; Fig. 1a,b). Second, to assess the unique and combined effects of warming, nitrogen addition, and grazing, we sampled a subset of the factorial temperature, nitrogen addition and grazing treatments in the *ThreeD* Global Change Experiment (referred to as the ***Global Change Experiment***; Fig. 1a,b). Third, to assess how warming responses vary across sites differing in precipitation we sampled a subset of the naturally occurring species in the ambient climate and warmed plots in three INCLINE Warming Experiment sites differing in precipitation (referred to as the ***Warming Experiment***; Fig. 1b,c).

In a field campaign conducted at the peak of the 2022 growing season we measured and computed functional traits (dataset i) related to plant and leaf size, leaf economics, and leaf nutrient status (morphological traits, including plant height and leaf wet mass, dry mass, area, thickness, specific leaf area [SLA], leaf dry matter content [LDMC]; and chemical traits, including carbon, nitrogen, and phosphorous content, C:N and N:P ratios, and d^13^C and d^15^N isotope ratios) from these systems. We also collected data on leaf assimilation-temperature responses (dataset ii), leaf spectral reflectance (dataset iii), leaf canopy temperatures (dataset iv), ecosystem CO_2_ fluxes (dataset v), landscape-scale and near-surface airborne multispectral imagery (dataset vi), and soil temperatures and soil moisture (dataset vii). In this paper, we first provide a basic description of the field sites and experimental designs and setups, before we describe the methods and data for each of the eight interconnected datasets presented here (Table 1). Note that the datasets vary in resolution and coverage within and across the three study systems, as detailed in the data descriptor below.

**Table 1:** Description and location of the datasets.

Dataset	Study system	Response variables	Number of data points^a^ and taxa^b^	Citation for raw data, clean data and code
i-a, i-b	Elevation Gradient Global Change Experiment Warming experiment	Plant functional traits	25,101 measurements^a^ 76 species^b^ 3,661 measurements^a^ 39 species^b^	Raw data^68^, clean data^68^, code^66^
ii	Elevation Gradient	Leaf assimilation-temperature responses	88 curves^a^ 3 species^b^	Raw data^81^, clean data^81^, code^82^
iii	Elevation Gradient	Leaf handheld hyperspectral readings	577 leaf spectroscopy readings 12 species^b^	Raw data^68^, clean data^68^, code^83^
iv	Elevation Gradient	Canopy leaf temperatures	2.26 billion raw temperature counts	Raw data^81^
v	Elevation Gradient Global Change Experiment	Ecosystem CO_2_ fluxes	1,323 flux measurements	Raw data^68^, clean data^68^, code^84^
vi-a, vi-b	Elevation Gradient Global Change Experiment	Landscape-scale airborne multispectral imagery	4,648 (x10 bands) individual multispectral images; 207 ground-truthing points	Clean data^68^
vii	Elevation Gradient Global Change Experiment	Microclimate	10,188 data points	Raw data^68^, clean data^68^, code^84^

This table summarises information on dataset number, response variable(s), number of observations, temporal range of the data, and location of the primary data, the final published data, and the code for extracting and cleaning data from the primary data. Superscripts refer to ^a^total number of observations in the data (i.e., data points), and ^b^number of taxa for which we have data. Note that chemical trait analyses are still in progress, and the planned final numbers are 8,574 measurements from 60 species. The OSF repository will be updated to include these measurements.

The datasets report on 28,762 plant and leaf functional trait measurements, 88 leaf assimilation-temperature response curves, 577 leaf handheld hyperspectral readings, 3,696 ecosystem CO_2_ flux measurements, and landscape-scale multispectral (10-band) and RGB cm-resolution imagery from airborne sensors, obtained from 4,648 individual images and covering four areas and a total area of 106,853 m^2^ (Table 1). While these data are from a relatively well-studied region where all vascular plant species we collected were already represented in public plant trait databases^30,31^, our contributions increased the number of unique trait measurements from this regional flora by 9%. Here, we present the data from this field campaign, along with the code to clean and integrate the datasets. Our aims are to safeguard the data for the future, expand global and regional trait data coverage, make data openly available, and facilitate future research.

The plant functional trait-based data reported here can be combined with extensive plant, vegetation, soil, and microclimate data from these study systems to allow exploration of the role leaf, community, ecosystem, or landscape response to experimental treatments or environmental gradients (for example^7,32^). This provides a valuable resource for testing hypotheses on biodiversity assembly, trait plasticity, trait filtering, ecosystem responses to global change, and vegetation-climate feedbacks. These data were collected during the sixth iteration of the Plant Functional Traits Course (PFTC6), an international training program in trait-based ecological theory and methods (https://plantfunctionaltraitscourses.w.uib.no/), see also^33^. The data aligns with information from similar courses and field campaigns conducted in China^34^, Peru^35^, Svalbard^36^, and South Africa (*in press*), paving the way for future comparative studies.

## Methods

### R packages

The data processing was mostly done in R^37^ and we used the R packages tidyverse^38^, tidylog^39^, janitor^40^, dataDocumentation^41^, dataDownloader^42^, data.table^43^, hms^44^, spectrolab^45^, broom^46^, PFTCFunctions^47^, osfr^48^, progress^49^, and writexl^50^ for data entry, data wrangling, and data cleaning. Analyses involved the packages LeafArea ^51^, rTPC^52^ and nls.multstart^53^. The packages ggplot2^54^, MetBrewer^55^ rcartocolor^56^, and scales^57^ were used for visualization. Automated setup and processing were carried out using targets^58^.

### Research site selection and general study setup

The data reported here were collected during the PFTC6 from July 7^th^ to August 1^st^ 2022 from seven semi-natural grassland sites in the fjord landscapes of western Norway (Fig. 1). Our research was conducted as part of three already established research projects in this study system: The Vestland Climate Grid is a grid of twelve grassland sites distributed across broad-scale temperature and precipitation gradients in western Norway, established in 2009^26,27,59^. The ThreeD Global Change Experiment was established along an elevational gradient with two Vestland Climate Grid sites in 2019. The INCLINE Climate Warming experiment was established in the four alpine Vestland Climate Grid sites in 2018^29^. All Vestland Climate Grid sites were selected to minimise variability in all factors other than climate, including vegetation type and structure, bedrock, slope, aspect, and land-use history^26^. All sites are on well-drained, relatively shallow soils, and on intermediate to calcareous bedrock. The target vegetation type was semi-natural boreal to alpine grassland vegetation^26^ corresponding to Nature in Norway (NiN 2.0) ecosystem types within T3 ‘mountain heath, lee-side, and tundra’ (the types T3-C-7 weakly calcareous lee-side, T3-C-10 strongly calcareous leeside) for alpine sites and T32 ‘seminatural grassland’ (several types, including T32-C-3, T32-C-4, T32-C-5, T32-C-13, T32-C-15) for sub-alpine and boreal sites (https://www.artsdatabanken.no/NiN). The current land-use regime is extensive free-range grazing by a mixture of domestic (goats, sheep, horses) and wild (deer, reindeer) grazers. Annual productivity ranges between 228–887 g m^−2^ y^−1^, while grazing pressure is generally low and ranges from 27.6–148.0 g m^2^ y^−1^, with higher values towards sub-alpine and boreal sites (AHH, *unpublished data*)^26–28^. Across the sites used for this study (see below), the mean annual precipitation (MAP) ranges from 1,256 to 3,601 mm/year and the mean growing season temperature, measured as the average of the four warmest months per year, ranges from 6.9 °C to 11.8 °C (Table 2).

**Table 2:** Overview over climatic, geographic, and research project information for each the seven study sites.

Site climate	Site name	Latitude (°N)	Longitude (°E)	Elevation (m a.s.l.)	MAP (mm)	MST (^°^C)	Study system	Research project
Boreal	Vikesland	60.8802	7.1699	469	1,292	11.8	Elevation Gradient Global Change Experiment	VCG ThreeD
North boreal	Høgsete	60.8760	7.1766	700	1,432	10.4	Elevation Gradient	VCG
Sub-alpine	Joasete	60.8618	7.1680	920	1,256	9.1	Elevation Gradient Global Change Experiment	ThreeD
Alpine	Liahovden	60.8599	7.1950	1,290	2,089	6.9	Elevation Gradient Global Change Experiment	ThreeD
Markedly oceanic alpine	Skjelingahaugen	60.9335	6.4150	1,088	3,601	7.9	Warming Experiment	VCG INCLINE
Slightly oceanic alpine	Gudmedalen	60.8328	7.1756	1,213	2,118	7.4	Warming Experiment	VCG INCLINE
Weakly oceanic alpine	Ulvehaugen	61.0243	8.1234	1,208	1,315	7.5	Warming Experiment	VCG INCLINE

The columns report on site bioclimatic zones and sections, site names, geographic coordinates (latitude, longitude), elevation (m a.s.l), mean annual precipitation (MAP), and mean summer temperature (MST, four warmest months, June-September), the study system to which the site belongs (for this paper) and connection to external research projects (for further information on experimental designs and additional data) for each of the seven sites. See text for details. Averages were calculated using daily means from 2008–2022 with data provided by the Norwegian Meteorological Institute (www.met.no). VCG = Vestland Climate Grid.

### Study design

#### The elevation gradient

This study system consists of four sites along an elevational gradient spanning from 469–1,290 m a.s.l. (Vikesland, Høgsete, Joasete, Liahovden) Fig. 1, Table 2). At each site, grazing exclusion fences were established, at the two lower sites as part of the Vestland Climate Grid in 2009 and at the two upper sites for the ThreeD project in 2019. We selected three plots inside and three outside these fences (i.e., six plots per site) to compare grazed versus non-grazed plant communities along elevation, and sampled plants and other data (see below) from these plots or on the site level. Further details of the gradient and sites are given in previous publications from the study system^26–29,59^.

#### The global change experiment

This study system uses the ThreeD project experiment which assesses the single and combined effects of three global change drivers (nitrogen deposition, warming, grazing) at three sites along an elevational gradient spanning 469–1,290 m a.s.l. (Vikesland, Joasete, Liahovden) (Fig. 1a, Table 2). Note that for this manuscript we sample and describe only a subset of plots and treatments from the full experiment. In 2019, ten experimental blocks were established at each of the three sites (Fig. 1a). Seven *nitrogen addition treatments*, ranging from 5–150 kg N ha^−1^ yr^−1^, and three replicates of a control (0 kg) treatment, were randomly allocated to these blocks, using the same randomization across all sites. Five of these treatments were used in this study; 0, 5, 10, 50, and 150 kg N ha^−1^ yr^−1^. For treatments we used oxidised nitrogen (NO and N_2_O), as this represents the main form of atmospheric nitrogen deposited in remote regions (i.e., away from intensive agriculture and other sources of reduced nitrogen), which we applied as slowly dissolving pellets (YaraBela OPTI-NS 27-0-0 (4S)) at the start and in the middle of the growing season from 2020–2022. At the alpine and sub-alpine sites, we established eight 50 × 50 cm plots within each block. These plots were given a unique number (origPlotID) between 1 and 160. We randomly allocated four of the plots within each block to a *warming treatment*, which was obtained by excavating and transplanting entire turfs to a lower-elevation site (sub-alpine and boreal, respectively), following established methods^25,27^. The transplanted turfs retained the same nitrogen treatment as the home site controls from the same block (obtained by transplanting into the same block number in the lower elevation site) and also obtained the destination plot ID of the plot into which they were transplanted (destPlotID). The turfs transplanted into the boreal Vikesland site were given destPlotIDs 161–200. The four replicate plots per nitrogen and warming treatment within blocks received different *grazing treatments*, of which two were used in this study: grazing exclosure (i.e., fencing) and natural grazing, see above. A fence was set up around the sites in spring 2020, leaving one ambient climate and one transplanted plot per block outside for the natural grazing treatment. Each plot was divided into a central 25 × 25 cm vegetation sampling zone and an outer zone used for destructive sampling. These corners of the outer and inner plots were marked with metal tubes. We sample plants and data (see below) in the destructive zone of the plots.

#### The warming experiment

This study system uses the INCLINE project Open Top Chamber (OTC) warming experiment replicated in four alpine sites, three of which are used here, located along a broad-scale precipitation gradient across the Scandes mountain range, capturing a difference of ca. 2,300 mm annual precipitation ranging from 1,315–3,601 mm yr^−1^(Ulvehaugen, Gudmedalen, Skjelingahaugen; Fig. 1c, Table 2, see^29^ for details). The field sites were fenced to prevent disturbance of the experimental infrastructure by animals and humans. At each site, seven pairs of OTCs and controls were established in 2018, placed to have comparable and homogeneous alpine grassland vegetation and comparable abiotic characteristics and contain two alpine target species of the INCLINE project (*Sibbaldia procumbens* and *Veronica alpina*), while avoiding rocks, depressions, and other features that made placement of OTCs difficult. Warming treatments randomised within each pair, with some adaptations as necessary due to practical constraints. The OTCs had a diameter of 1.5 m and a height of 40 cm and were constructed following the general ITEX protocol^60^. To avoid damage by heavy snow they were stored on-site during winter and only placed on the plots during the snow-free period each year^29,61^. Within each OTC and control, several 25 × 35 cm experimental plots were established, see^29^ for details. We sample plants and data (see below) inside and outside OTCs, avoiding the INCLINE experimental plots and focal plants.

### Background and other datasets

**Fig. 2:**
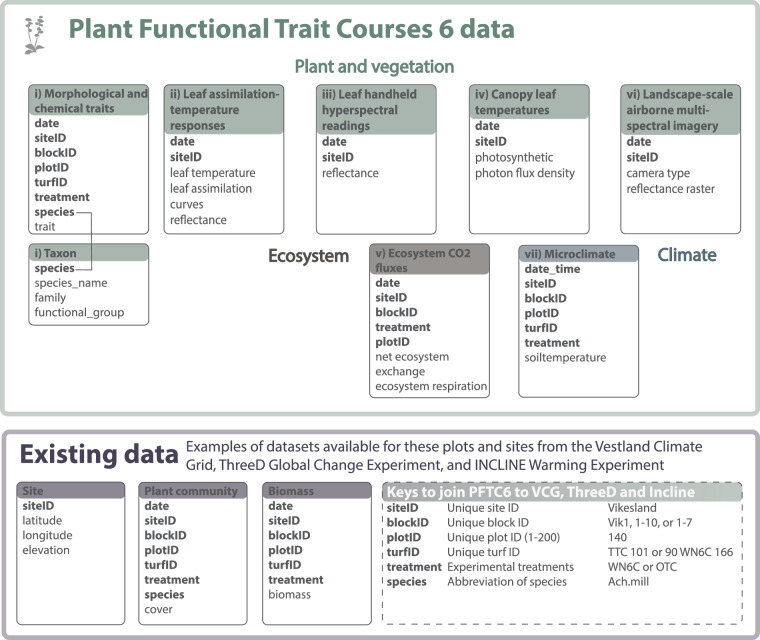
Data structure for the plant functional trait and associated datasets. Boxes represent the data tables for plant functional traits (dataset i), leaf assimilation-temperature responses (dataset ii), leaf handheld hyperspectral readings (dataset iii), canopy leaf temperatures (dataset iv), ecosystem CO_2_ flux (dataset v), landscape-scale airborne multispectral imagery (dataset vi), and microclimate (datasets vii). Names of individual data tables are given in the coloured title area and a selection of the main variables is available within tables in the internal lists. For full sets of variables, see Tables 3, 4, 6–9. All bold variables are shared between several tables and can be used as keys to join them. For example, the line linking the plant trait and taxon boxes exemplifies how the variable ‘species’ can be used to join these two tables. As our study systems were established within existing projects and experiments, the data presented here can also be linked with extensive datasets from these projects on e.g., environment and climate, plant community composition, cover, biomass, fitness, and reproduction. Examples of existing data that are especially relevant for the data reported in this paper are given in the “Existing data” box at the bottom row. Keys to link the data reported in this paper with the Vestland Climate Grid, ThreeD, and INCLINE projects are given in the bottom hatched box, with an example value for each variable on the right.

All sites except Joasete and Liahovden are part of the Vestland Climate Grid, established in 2009, from which additional data exist on site-level environmental, climate, soil chemical, soil structure, and plant functional trait data, and plot-level litter decomposition, Teabag Index^62^ decomposition, ecosystem carbon flux, and microclimate data; and plot-level long-term records of vegetation composition, cover, biomass and performance from climate change and functional group removal experiments (see e.g.,^26–28,59^ for details). The Skjelingahaugen, Gudmedalen, and Ulvehaugen sites are also part of the INCLINE project, established in 2018, from which additional data exist on site-level climate and soil data and plot-level records of plant performance, vegetation composition, and ecosystem carbon fluxes from warming and lowland plant invasion experimental plots^29^. The Vikesland, Joasete, and Liahovden sites are part of the ThreeD project, established in 2019, from which additional data exist on site-level environmental, climate, and soil chemistry, soil structural data; plot-level Teabag Index decomposition rates, ecosystem carbon fluxes; and plot-level long-term records of microclimate, vegetation composition, cover, biomass and life-history data from warming, grazing, and nitrogen deposition experimental plots (see^63^ for details). The existing long-term vegetation composition records are of particular relevance here. These data consist of plot-level data on vegetation cover, vegetation height, and percent cover of each species, and sub-plot level data on plant performance (life-history stage, size, fertility) of each vascular plant species in each 5 × 5 cm subplot from 2009–2024 (Vestland Climate Grid), 2018–2023 (INCLINE) and 2019–2022 (ThreeD). Repeated measures allowed checking of the data for consistency over time by comparing sub-plot data across years, see^27^ for details on methods and data. These data are available in the Vestland Climate Grid^64^, ThreeD^63^, and INCLINE^65^ OSF repositories and can be linked to the data described here through various keys, including species, sites, and plots (see Fig. 2), which allow the combination of vegetation composition and trait data for community-weighted trait distribution analyses. Examples of code to access and download relevant datasets from these repositories is provided in the code^66^.

### Species identification, taxonomy, and flora

All vascular plant specimens collected during the PFTC6 campaign from July 7^th^ to August 1^st^ 2022 were identified to the species level in the field, with nomenclature following Lid and Lid^67^. Exceptions were sterile specimens of species that cannot be easily identified without reproductive parts (for example, *Alchemilla* spp. excluding *A. alpina*, and *Euphrasia* spp.). All such specimens were included and flagged in the dataset, as described below. The full species names, taxon names of taxa not identified to the species level, are provided in the taxon table on OSF^68^ (Fig. 2).

### Dataset collection methods

#### Dataset (i-a and i-b): plant functional traits

##### Sampling designs

We measured plant functional traits across all experiments and sites. Specific sampling designs, described below, were aimed at assessing community trait distributions (Elevation Gradient and Global Change Experiment, dataset i-a), intraspecific trait variability (targeting 16 species from the Warming Experiment, dataset i-b), with additional samples analysed as needed to link to datasets ii – vi (see below).

##### Global change experiment and elevation gradient

Within each plot, we sampled up to five leaves from the most abundant species in that plot. These species were pre-selected based on the existing community composition data described under ‘Background and other datasets’ above (see also Fig. 2). The number of species per plot varied as we aimed to sample species that collectively sum up to at least 80% of the total observed cover of each plot. Individuals of the same species were sampled as far apart from each other as possible within the plot to avoid sampling multiple ramets of the same genet, and, for the Global Change Experiment, they were sampled in the outer destructive zone of the plot, avoiding the core area (see above).

##### Warming experiment

Within each block × treatment (i.e., OTC or Control) we sampled one leaf each from a minimum of three and up to nine individuals of all species present from a list of 16 pre-selected target species. The target species were selected to occur across all Warming Experiment sites with additional species sampled to increase the community trait coverage and overlap with the Climate Gradient and Global Change Experiment, for a total of up to 38 species per site. Four blocks (pairs of OTC and Control treatments) were sampled at Gudmedalen and Skjelingahaugen. At Ulvehaugen, five blocks were sampled because the blocks are spread across a valley with opposing aspects as we wanted replicates from each aspect. For each target species present in a specific treatment plot, we selected individuals at least 50 cm apart where possible to avoid sampling multiple ramets from the same genet. We did not sample close to the OTC margins to avoid possible edge effects, and we avoided sampling in the INCLINE project long-term monitoring plots.

Additionally, plant functional traits as described below were also collected for the leaves measured for leaf-level AT responses (three species from along the Elevation Gradient, dataset ii), leaf handheld hyperspectral readings (12 species from along the Elevation Gradient, dataset iii), four dominant species from each of the canopy leaf thermal imagery plots (dataset iv), and landscape-scale airborne multi- and hyperspectral imagery (Elevation Gradient, links to dataset vi). These leaf trait measurements are reported within these respective datasets.

##### Field measurements and leaf or plant collection

For all trait measurements, we selected reproductively mature adult plant individuals when possible. For each individual, we measured vegetative plant height in the field as the shortest distance between the ground and the highest photosynthetic tissue on the plant, measured when the plant was in its natural form (i.e., not stretched height) and excluding reproductive structures such as buds, fruits, or flowers. We then sampled fully expanded leaves from these individuals, avoiding damage and disease, when possible, and placed the these into an individual Ziploc^TM^ bag per plant individual, with a damp paper towel to keep leaves hydrated. The leaves were transported to the lab, placed in the fridge and processed for the traits described below within 24 (in a few cases, up to 48) h. All trait measurement protocols are based on and adapted from the guidelines by Pérez-Harguindeguy *et al*.^69^.

*Plant functional trait measurements and calculations*

1. *Leaf processing* - we typically selected one to three healthy and fully expanded leaves per individual for further processing, except for species with very small leaves where we included multiple leaves per sample in order to obtain a leaf dry mass of at least 0.03 g (the mass needed for chemical traits analysis; see below). For *Selaginella selaginoides*, we sampled five sterile branchlets. For *Calluna vulgaris*, we sampled five of the previous year’s short shoots, following^70^. For forb and woody species, leaf samples included the petiole, rachis, and stipules when present. For graminoids we sampled the leaf blade without the leaf sheath. Leaf samples were carefully trimmed according to these criteria and pat-dried with a paper towel to remove excess water. Each leaf or multiple-leaf sample was then placed back in the Ziploc^TM^ bag. For each leaf sample, a unique leaf ID with an associated barcode was assigned, and the number of leaves in composite samples, the vegetative height, and leaf and plot ID data were recorded on a purpose-made envelope label glued on a coin envelope. This envelope was stapled to the individual Ziploc^TM^ bag for the duration of the trait measurements to ensures that leaves were moist and uniquely identified during trait measurements. Data on trait measurements were recorded on the envelope label.
2. *Wet mass* - Wet mass of each leaf sample was measured to the nearest 0.0001 g using Sartorius CP224S and BP221S scales (0.1 mg precision). Before weighing, any excess water was removed by patting the leaf surface dry with a paper towel. The leaf sample was placed back in the Ziploc^TM^ bag after weighing, and wet mass was recorded on the envelope label.
3. *Leaf area* - Each leaf sample was scanned using a Canon LiDE 220 scanner. Before each scan, the scanner was cleaned of water or debris. Any excess water was removed from the leaf by patting the leaf surface dry with a paper towel, and the leaf, still at field turgor, was placed flat on the scanner face-down, ensuring the leaf was not folded and the leaflets did not overlap (if possible). We used transparent tape to tape leaves or leaflets to the glass surface as needed. The leaf was placed away from the scanner edge to ensure that the entire leaf was visible in the scan. The graminoids *Festuca rubra, F. ovina, F. vivipara, Avenella flexuosa*, and *Nardus stricta* have naturally very tightly folded leaves and were left folded during scanning. During data analysis, the scanned area for these species was multiplied by two to obtain the correct total leaf area. Large leaves were carefully cut into pieces for scanning, with the number of pieces recorded on the envelope label. The image quality was checked for each scan. The leaf ID and scanning settings were automatically checked. If the quality was insufficient or the labelling or settings were incorrect, the image was deleted, the error corrected, and the leaf scanned again. The number of leaves (for multiple-leaf samples) or leaf pieces (for large leaves) was manually checked against the data on the envelope label after scanning. If the leaf number was correct, the leaf sample was placed back into the Ziploc^TM^ bag and proceeded to the next station, else, the leaf was returned to the weighing station for wet mass correction. Leaf area was calculated from each scan using ImageJ^71^ and the Leaf Area package^51^.
4. *Leaf thickness* - For each leaf sample, three thickness measurements were taken at various points on the lamina using a Micromar 40 EWR digital micrometre, avoiding thicker parts of the leaf-like midrib if possible (i.e., unless the leaf was too small). For small leaves, where repeated measurements would have overlapped, less than three measurements were taken.
5. *Data Entry* - The data from the envelope label of each leaf sample was checked and digitised into a spreadsheet, using drop-down menus as appropriate for values (e.g. for species names) to avoid typing errors. If any information was missing and the leaf was still fresh enough for the measurements, the leaf sample was returned to the appropriate trait measurement station for completion. Each envelope was photographed for documentation and to allow any errors during data entry to be corrected. The leaf or leaves were then placed into the labelled coin envelope.
6. *Dry mass* – The coin envelopes containing the leaves were dried at 65°C for 72 h before leaf samples were weighed, using the same balances as above. After weighing, the number of leaves (for multiple-leaf samples) or leaf pieces (for large leaves cut into pieces for scanning) was again checked against the data on the envelope, any errors were noted, and the leaf sample was placed back into the coin envelope. This station completes the leaf trait analyses during the field campaign.
7. *Chemical traits measurements* - A subset of leaves was transported to the University of Arizona for analyses of leaf carbon, nitrogen, phosphorous, and the isotopes δ^15^N, and δ^13^C. Each leaf was ground to fine powder. If single-leaf samples had insufficient dry leaf material for analysis, leaves from the same site and treatment were pooled. To determine the total phosphorous concentration, each sample was treated with persulfate oxidation and the acid molybdate method^72^ and then measured colorimetrically with a spectrophotometer (Thermo Scientific Genesys20). Leaf carbon and nitrogen concentration and their stable isotope ratios were analysed by the Geosciences Environmental Isotope Laboratory at The University of Arizona. Samples (1.0 ± 0.2 mg) were combusted on a continuous-flow-gas-ratio mass spectrometer (Finnigan Delta PlusXL) and processed with a Costech elemental analyser. The data were standardized using acetanilide for N and C concentration, NBS-22 and USGS-24 for δ13C, and IAEA-N-1 and IAEA-N-2 for δ15N. Ratios between C:N and N:P were also calculated and analysed.
8. *Data processing -* In cases where we used multiple leaves from one individual plant for one leaf sample, we divided the wet mass, dry mass, and total leaf area by the number of leaves to calculate average trait values per leaf. For some samples, leaves had been lost between the wet and dry mass measurement, and for those we divided the dry mass by the appropriate number of leaves at that station. Leaf thickness was calculated by taking the mean of the individual measurements. Specific leaf area (SLA) was calculated by dividing leaf area by dry mass and leaf dry matter content (LDMC) by dividing dry mass by wet mass, using average trait values per leaf for samples based on multiple leaves. Note that the trait data from the Elevation Gradient and Global Change Experiment (dataset i-a) and the Warming Experiment (dataset i-b) are split into two different datasets. Also, note that within each of these datasets, morphological and chemical traits are reported in separate data tables, and that chemical traits only exist for a subset of individuals. In cases where pooled leaf samples were used for leaf chemical trait analyses, the resulting data values are reported for all individuals contributing to the pooled sample. For guidance on how to merge and use morphological and chemical traits, see Usage Notes.

#### Dataset (ii): Leaf assimilation-temperature responses

We measured leaf assimilation-temperature responses of four target species (*Agrostis capillaris*, *Alchemilla alpina*, *Achillea millefolium, and Vaccinium vitis-idaea*) sampled from the Elevation Gradient sites (Fig. 1a).

##### Field sampling of turfs

Plant material was acquired by cutting turfs from the Elevation Gradient sites (Fig. 1a) and transporting them to the laboratory for measurement. To minimise disturbance to the root systems of our plants, we cut turfs with a minimum size of ca. 30 cm by 30 cm, to a depth of ca. 15 cm or to the bedrock, whichever was shallower. Turfs were given a numeric ID, covered with a black plastic bag, placed in waterproof containers and immediately transported to the laboratory. They were kept well-watered in a sun-exposed location outdoors when not in use. Turfs were typically measured within 12 h of collection, but up to a maximum of 72 h in some cases.

##### Lab measurements of assimilation-temperature response

In the lab, we measured assimilation-temperature responses at the leaf level on our collected plant material (see below). All measurements were carried out on LI-6800 Portable Photosynthesis System gas exchange analysers (LI-COR Biosciences Inc., Lincoln, NE, USA).

We estimated saturating photosynthetic photon flux density (PPFD) for each of our species before measuring assimilation-temperature responses. We measured light response curves following Heberling & Fridley^73^ on three individuals of each species. Leaf temperatures were held at 20 °C, with airflow rate set to 600 µmol s^−1^, relative humidity set to 35%, and reference CO_2_ set to 420 µmol mol^−1^. PPFD was then decreased from 1,800 to 10 μmol m^−2^ s^−1^ in nine steps. We then fit a light response model^74^ and estimated the PPFD value, which gives 80% of the maximum assimilation rate. The mean values for each species (185 μmol m^−2^ s^−1^ for *Alchemilla alpina*, 359 μmol m^−2^ s^−1^ for *Achillea millefolium*, 433 μmol m^−2^ s^−1^ for *Vaccinium vitis-idaea*) were used in subsequent assimilation-temperature measurements. We obtained few clean assimilation-temperature curves from *Agrostis capillaris* and thus dropped this species from these analyses.

We measured assimilation-temperature response using the Fast Assimilation-Temperature Response (*FAsTeR*) method^75^. Briefly, leaves were exposed to linearly increasing temperature conditions in the LI-6800 cuvette, and high-frequency nonequilibrium measurements of leaf temperature and assimilation were conducted. Leaves were placed into the cuvette when internal cuvette air temperature was roughly equal to ambient temperature, ensuring that the abaxial leaf surface made good contact with the leaf temperature thermocouple. PPFD was set to the previously determined saturating value, and relative humidity was maintained at 35%. Leaves were allowed to acclimate to the cuvette environment for at least 20 min while heat exchanger temperatures were cooled to the minimum achievable values (−1 to 14 °C, dependent on ambient conditions). A linear temperature ramp was then executed spanning 40 °C starting at the lowest achievable heat exchanger temperature at a rate of 1.5 °C min^−1^, for a total ramp time of 30 min. Airflow rates were set to 250–300 μmol s^−1^, and fan speed was maintained at 10,000 RPM. While the temperature increased, data were logged in 2 s intervals with 1 s signal averaging for a total of 33 min. After measurements were complete, the portion of the leaf enclosed in the cuvette was marked with a pen, and the marked portion of the leaf was cut out and scanned with a flatbed scanner at 300 dpi. The area of the measured leaf portion was then calculated using ImageJ^71^ and the LeafArea package in R^51^, and assimilation values were then recalculated using the measured leaf area. Post-measurement corrections for nonequilibrium effects and gas analyser drift were applied by adapting *FAsTeR* code, following^75^.

#### Dataset (iii): Leaf handheld hyperspectral readings

Plant material from turfs collected for leaf assimilation-temperature response (dataset ii) were paired with spectroscopy measurements (SVC HR 1024i with a LC-RP PRO attachment; 350–2400 nm; Spectra Vista Corporation). We measured reflectance on a single individual belonging to the dominant species in each quadrant. The magnetic jaw on the leaf clip was removed to use the LC-RP PRO as a probe and pressed firmly onto the target individual leaf. Two to three measurements were taken for each leaf and visualised instantly with SVC HR-1024i Data Acquisition Software (version 1.22.26). Any extraneous measurements were flagged for removal. All spectroscopy measurements were made following leaf temperature and gas exchange measurements (within less than five hours).

#### Dataset (iv): Canopy leaf temperatures

We measured *in situ* leaf temperatures and microenvironmental data in the grazed vegetation at each of the Elevation Gradient sites (Fig. 1a) using a custom-built thermal imaging camera apparatus using a FLIR A700 thermal infrared imaging camera controlled by a Raspberry Pi microcomputer based on Blonder *et al*.^76^. The camera was controlled using software modified as described in Blonder *et al*.^76^ (https://github.com/bblonder/flir_thermal_control) to incorporate the A700 and other peripheral sensors described below. The camera was mounted at a height of 1.7 m, oriented perpendicular to the ground. The field of view was ca. 1.4 × 1.1 m at a resolution of 640 × 480 pixels, for a pixel size of ca. 2 × 2 mm. The camera includes a visual image camera and was configured to capture a thermal image and visual image once every 5–6 s from sunrise to sunset (ca. 5:10 to 22:20), with each site sampled on a different date between July 27^th^ and 31^st^ 2022. The thermal imaging camera apparatus included peripheral sensors for gathering microenvironmental data. Ambient air temperature was measured by a bare-wire T-type copper-constantan thermocouple housed in a Stevenson screen. Soil temperature was measured by a sheathed T-type thermocouple (Omega Engineering Inc.) placed at a depth of 10 cm. PPFD was measured using a LI-COR LI-190R quantum sensor. Microenvironmental data were acquired at the same frequency as the thermal imaging. A black reference plate with an embedded thermocouple was placed in the camera’s frame for ground truth calibration^76,77^.

#### Dataset (v): Ecosystem CO_2_ flux data and flux calculations

We measured ecosystem CO_2_ fluxes at hourly intervals over a full diurnal cycle (24 h) during peak growing season (July 23^rd^ - 31^st^, 2022) along the Elevation Gradient and in the Global Change Experiment. In both systems we focused on effects of temperature, comparing ungrazed and unfertilised plots along the Elevation Gradients and the warmed vs. ambient climate treatments in the Global Change Experiment. Measurements were done at three replicate plots per treatment and site, for a total of *n* = 6 warmed plots and *n* = 12 ambient plots across sites.

For each plot and measurement time, we first measured Net Ecosystem Exchange (NEE) using the transparent chamber, and we then measured Ecosystem Respiration (ER) by covering the chamber with a dark cloth blocking the sunlight. Gross Primary Production (GPP) was later calculated from these measurements (see below for details on field measurements and calculations).

##### Field measurements

Each of the flux measurements were made using a closed loop plexiglass chamber system connected to a gas analyser (IRGA; Li-840, LI-COR Biosciences, Lincoln, NE, USA) measuring CO_2_ concentration at 1 Hz. The chamber (25 × 25 × 40 cm) matched the size and height of vegetation in the experimental plots. At each measurement, the base of the chamber was sealed onto the plot by a tarp and heavy chain to prevent air leakage. A fan mounted on the chamber wall ensured air mixing within the chamber, an air pump ensured a flow of 1 L min^−1^ through the IRGA, and a filter at the start of the incoming air tube prevented water droplets and small particles from entering the IRGA. The chamber was also equipped with thermocouples (Pt1000, Delta-T) to measure air and soil temperatures (soil temperature placed at 2 cm depth), and a photosynthetically active radiation (PAR) LI-190/R Quantum sensor (LI-COR Biosciences) connected with a millivolt adapter. Environmental data (PAR, and soil and air temperature) were measured at 10 seconds intervals. All data from the IRGA, thermocouples and PAR sensor were logged with a Squirrel Data logger 2010 series (Grant Instruments).

The chamber was aired for 1 min before each measurement to prevent the accumulation of CO_2_ in the chamber and tubes. The starting time for each measurement was recorded manually, and CO_2_ concentration was recorded for 180 s. This duration also mitigated the influence of increasing temperature on the plants within the chamber.

##### Data processing and calculations

The first and last 10 s of each flux measurement were removed. Then, each flux was fitted to an exponential function^78^:

1 $$C\left(t\right)={Cm}+a\left(t-{tz}\right)+\left({Cz}-{Cm}\right){e}^{-b\left(t-{tz}\right)}$$

Where *C(t)* is the CO_2_ concentration as a function of time, *Cm* is the CO_2_ concentration when equilibrium is established in the chamber, *a* and *b* are fitting parameters, *Cz* is the intercept of the linear fit of the first 15 s of the flux and *tz* is defined as C(tz) = Cz. To estimate *Cm*, *a*, *b* and *tz* we fitted the CO_2_ time series of the observed CO_2_ in an iterative way using the optimization function (base::optim) with default parameters in R^37^ on the root mean square error (RMSE) for each flux^78^.

To calculate the flux, the slope at *tz* is then used:

2 $${C}^{{\prime} }\left({tz}\right)=a+b\left(Cm-Cz\right)$$

The flux of CO_2_ is calculated as follows:

3 $${NEE}=\left(\frac{\partial {{CO}}_{2}}{\partial t}\right)\times \frac{P\times V}{R\times T\times A}$$

Here, in Eq. 3, the *flux* is the flux of CO_2_ at the surface of the plot (mmol m^−2^ h^−1^),

where $\frac{\partial {{CO}}_{2}}{\partial t}$ is the slope of *C(t)* (ppm s^−1^), *P* is pressure (assumed 1 atm), *V* is the volume of the chamber and tubing (L), *R* is the gas constant (0.082057 L*atm*K^−1^*mol^−1^), *T* is the chamber air temperature (K), and *A* is the area of the chamber frame base (m^2^).

We calculated GPP as:

4 $${GPP}={NEE}-{ER}$$

where GPP is negative to indicate CO_2_ removal from the atmosphere, and ER is positive to indicate CO_2_ addition to the atmosphere.

#### Dataset (vi-a and vi-b): Landscape-scale airborne multispectral imagery

##### Multispectral (10-band) imagery

**Fig. 3:**
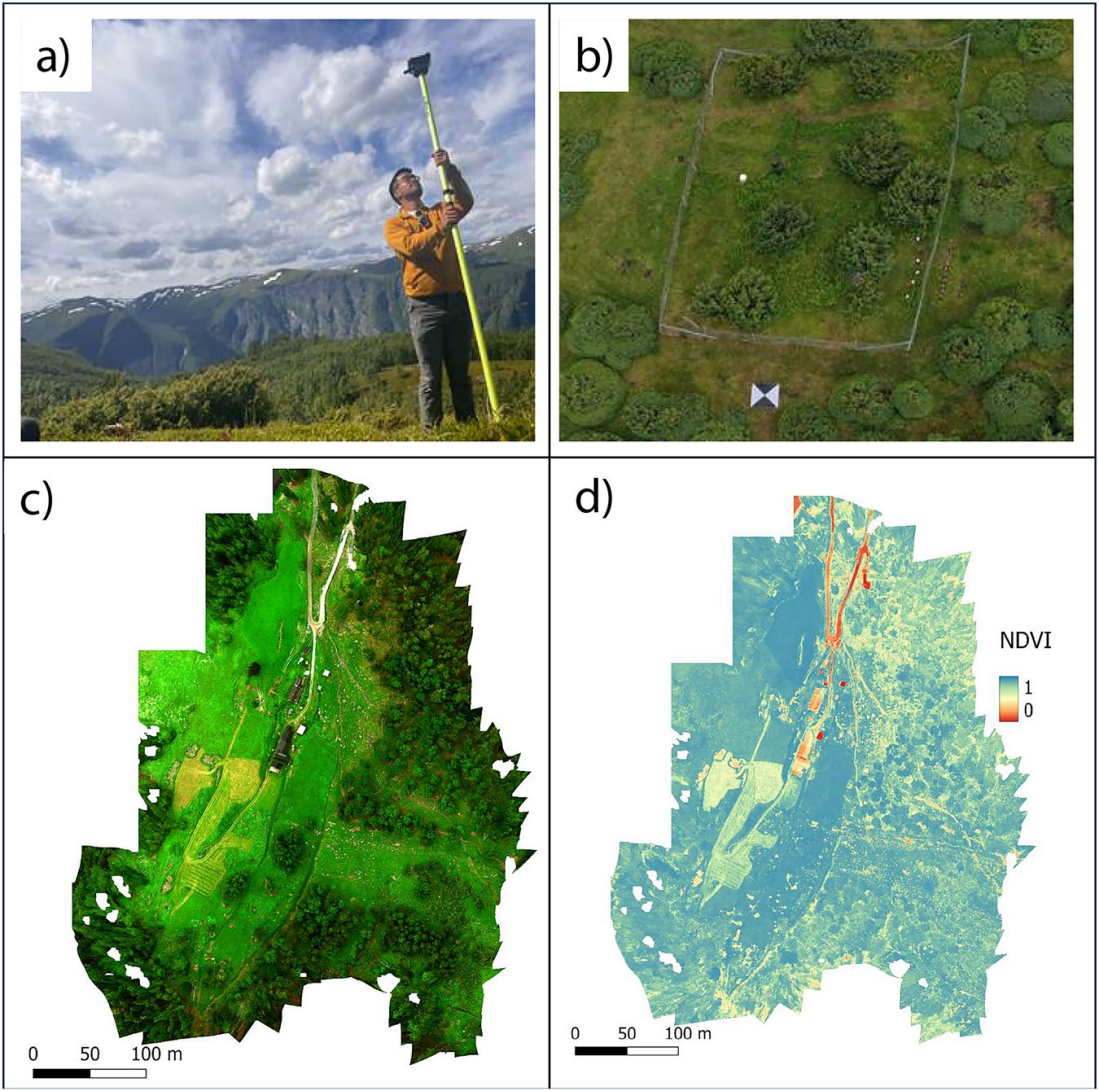
Illustration of methods and outputs for landscape-scale airborne multispectral imagery. (**a**) Mapping Ground Control Points (GCPs) using Emlid Reach RS + differential GNSS system; (**b**) GPC view from the drone at the north boreal site; (**c**) true colour orthomosaic representing the lower section of the boreal site; (**d**) orthomosaic for the same area representing the Normalised Difference Vegetation Index (NDVI).

A 10-band MicaSense Dual Camera (AgEagle Aerial Systems Inc., Wichita, Kansas, USA) mounted on a DJI Inspire 2 (SZ DJI Technology Co., Shenzhen, Guangdong, China) was flown at each of the four Elevation Gradient sites (three of which are also in the Global Change Experiment; see Table 2). The 10 reflectance bands are: Coastal Aerosol [444 ± 28 nm], Blue [475 ± 20 nm], Green [531 ± 14 nm and 560 ± 20 nm], Red [650 ± 16 nm and 668 ± 10 nm], Red Edge [705 ± 10 nm, 717 ± 10 nm, and 740 ± 15 nm], and Near Infrared [840 ± 40 nm]. Ten to 12 ground control points (GCPs) were placed on each site ahead of the flight and georeferenced using an Emlid Reach RS + differential GNSS system to an accuracy of <3 cm (Fig. 3a). Further, 207 patches of dominant vegetation were also georeferenced across the sites, targeting 38 locally dominant vascular plants, bryophytes, and lichen species, plus two land cover types not taxonomically identified (“crust” and “moss”). The drone missions were planned and executed using the Atlas Flight app (AgEagle Aerial Systems Inc.). Flights were performed over a range of elevations from 30 to 80 m above the ground to adapt for the steep terrain and with an image overlap of 75%.

For each of the four sites along the Elevation Gradient (Fig. 1a), the individual overlapping images, together with the GCPs, were processed using Agisoft Metashape Professional (v1.7.1) using an *structure-from-motion* (sfm) workflow following the recommendations of Over *et al*.^79^ to create very high resolution orthomosaics for each of the 10 reflectance bands. Camera calibration involved the MicaSense Dual Camera parameters (i.e., focal length, principal point offset, radial distortion coefficients, tangential distortion coefficients, skew). Bundle adjustment consisted of removing all tie points with a reprojection error >0.5 and reconstruction uncertainty >20%.

##### RGB (true colour), very high-resolution imagery

For the two lower-elevation sites within the Elevation Gradient (Vikesland, Høgsete), additional RGB flights were performed with a DJI Mavic 2 Pro drone (SZ DJI Technology Co., Shenzhen, Guangdong, China) equipped with a Hasselblad L1D-20c camera (Victor Hasselblad AB, Gothenburg, Sweden), which produces 5472 × 3648 pixel images (20MP). These flights, flown at the same elevations and over the same areas as the ones done with the DJI Inspire 2 drone but with less overlap, aimed to produce extremely high RGB reference orthomosaics from 370 images in Vikesland and 879 images in Høgsete. GCPs were produced and georeferenced as described for the 10-band imagery (11 GCPs each for Vikesland and Høgsete; see section Data Records, Dataset (vi) for more detail). The DJI Mavic 2 Pro stopped working towards the end of data collection and this precluded flights over the two higher elevation sites within the Elevation Gradient (Joasete, Liahovden). For the Vikesland and Høgsete sites, the individual overlapping images, together with the GCPs, were processed using Agisoft Metashape Professional (v1.7.1) and followed the same procedure as with the 10-band imagery to create very high resolution RGB reference orthomosaics. Camera calibration involved the Hasselblad L1D-20c camera parameters.

##### Photogrammetry flight

An experimental flight was done in a subsection at Høgsete dominated by *Juniperus communis* shrubland, which contained a fenced area precluding sheep grazing (Fig. 3b). The objective of this flight was to test the *structure-from-motion* (sfm) photogrammetric technique and produce a dense 3D point cloud from highly overlapping images obtained from the drone cameras flown at different angles. We flew at an elevation of 30 m using the Hasselblad L1D-20c camera mounted in the DJI Mavic 2 Pro. Five GCPs were placed and georeferenced as described in the previous two sections. 224 images were produced with an overlap of 80% over an area of 0.01 ha encompassing grazed and ungrazed juniper shrubland. The images resulted from two flights: the first one done with the camera at nadir and second one done with the camera at an angle 20° off nadir. The drone mission was planned and executed using the Atlas Flight app.

The individual overlapping images, together with the GCPs, were processed using Agisoft Metashape Professional (v1.7.1) using the same sfm workflow than with the 10-band imagery above. Geometric calibration used the five geo-referenced GCPs and the 224 images. Camera calibration involved the Hasselblad L1D-20c camera parameters.

#### Dataset (vii): Microclimate

We recorded microclimate data in the ungrazed plots at the four sites along the Elevation Gradient and the control and warmed plots (ungrazed and unfertilized) in the Global Change Experiment (Fig. 1a), paralleling the ecosystem carbon fluxes measurements (see dataset v above). TMS-4 loggers (TOMST) were installed next to each plot between August 22^nd^ 2019 and July 29^th^ 2022, recording near-surface, ground, and soil temperature (15, 0, and −8 cm), and soil moisture every 15 min. Loggers were removed either after the completion of the flux measurements or after the completion of the experiment, between July 28^th^ and September 2^nd^ 2022. We calibrated the raw soil moisture signal following Appendix A in Wild *et al*.^80^ using the soil type silt-loam which was the most appropriate for these soils (Aud Halbritter, *unpublished data*). Additionally, we measured *in situ* leaf temperatures and microenvironmental data as described above (dataset iv)). Additional site- and plot-level environmental and climate data are available from the Vestland climate Grid, ThreeD, and INCLINE projects (see above, and Fig. 2)^80^.

## Data Records

This paper reports on plant functional traits and associated data on leaf and ecosystem carbon fluxes, and thermal, multi- and hyperspectral imagery from three study systems; an Elevation Gradient, a Global Change Experiment, and a Warming Experiment. The data were collected in boreal, sub-alpine, and alpine grassland vegetation in western Norway by the PFTC6 plant functional traits course during peak growing season in 2022 (Fig. 1).

Data outputs consist of seven datasets. The core dataset (i) consists of plant functional trait data representing the plant community of the Elevation Gradient and Global Change Experiment, and 16 preselected focal species plus additional dominant species across the four Warming Experiment sites, along with additional trait measurements made to match traits to the other datasets collected. At the leaf-level, we report on (ii) leaf assimilation-temperature responses, (iii) leaf handheld hyperspectral readings, and (iv) canopy leaf temperatures from the Elevation Gradient. At the ecosystem level, we report on (v) diurnal ecosystem carbon fluxes from control and warmed plots in the Global Change Experiment and along the Elevation Gradient, (vi) landscape-scale airborne multispectral imagery covering the Global Change Experiment and Elevation Gradient, and (vii) microclimate data from control and warmed plots in the Global Change Experiment and along the Elevation Gradient (Table 1). Each dataset includes the focal response variable(s) (i-vii) along with associated study design, global change treatment, and climate variables (Fig. 2, Table 1).

### Data organization and structure

The final clean data files are available on OSF^68,81^. All files are named using the following naming structure: nr_PFTC6_clean_experiment_variable_year(s).csv. The nr refers to the roman dataset number in Table 1; experiment refers to the Elevation Gradient, Global Change Experiment or Warming Experiment; the variable corresponds to the response variable using the terminology in Table 1. All datasets are structured similarly, sharing some common variables including year, date, siteID, blockID, plotID, turfID, and treatments and specific variables that are unique to each dataset (Fig. 2). The shared variables can be used to link different datasets, for example to combine them for specific analysis (bold letters in Fig. 2).

The code necessary to access the raw data and produce cleaned datasets, along with explanations of the various data cleaning steps, issues, and outcomes, are available in open GitHub repositories, with versioned copies archived in Zenodo^66,82–84^. The raw data files are available at Open Science Framework (OSF)^68,81^ in a folder called “raw_data”. In this folder there is a separate folder for each dataset, containing several raw data files. The folder is named using the roman letter corresponding to Table 1. The Usage Notes section in this paper summarises the data accuracy and data cleaning procedures, including explanations of and advice on how to deal with various comments and flags in the data, caveats regarding data quality, and our advice on ‘best practice’ data usage. The reader is referred to the code and the detailed coding, data cleaning, and data accuracy comments and the associated raw and cleaned data and metadata tables below for further information.

### Dataset (i-a, i-b): Plant functional traits

Tables 3 and 4 describe all variables in the plant functional trait datasets. Note that due to different sites and experimental designs, the data are reported in two tables, i-a contains Elevational Gradient and Global Change Experiment data, i-b contains the Warming Experiment data. The file *R/trait_plan.R* in the GitHub repository^66^ and the specific functions referred to in this script provides the code to download and clean the data.

**Table 3:** Data dictionary for plant functional traits from the Elevation Gradient and the Global Change Experiment (dataset i-a).

Variable name	Description	Variable type	Variable range or levels	Units	How measured
ID	Unique leaf ID	categorical	AAB3003 - IPJ1358		defined
date	Sampling date	date	2022-07-24 - 2022-08-01	yyyy-mm-dd	recorded
gradient	Sample from the elevational gradient	categorical	gradient		recorded
siteID	Unique destination site ID	categorical	Hogsete - Vikesland		defined
elevation_m_asl	Site elevation	numeric	469–1290	m a.s.l.	recorded
blockID	Unique destination block ID	numeric	1–10		defined
turfID	Unique turf ID, as origPlotID, treatments, destPlotD	categorical	105 WN3C 173 - 91 AN6C 91		defined
warming	Warming treatment with W for warming or A for ambient	categorical	A - W		defined
grazing	Grazing treatment with N for natural grazing and C for control	categorical	C - N		defined
Nlevel	Nitrogen treatment levels 0–10	numeric	0–10		defined
Namount_kg_ha_y	Amount nitrogen added	numeric	0–150	kg ha^−1^ y^−1^	defined
individual_nr	Individual number	numeric	1–10		defined
species	Scientific name	categorical	*Achillea millefolium* - *Viola tricolor*		identified
trait	Plant functional leaf trait	categorical	dry_mass_g - wet_mass_g		defined
value	Trait value	numeric	0–500	cm, g, cm^2^, mm, cm^2^ g^−1^, %, ‰	recorded
origSiteID	Unique site ID of origin site	categorical	Joasete - Liahovden		defined
destSiteID	Unique site ID of destination site	categorical	Joasete - Vikesland		defined
comment	Comment on the data	categorical			recorded
problem	Describing the issue for flagging data	categorical	blockID and grazing imputed - wet or dry mass might be wrong		recorded
flag	Flagging missing or unreliable data	categorical	missing dry mass - unreliable wet mass		recorded

Data and variable descriptions for dataset i-a – plant functional traits along an elevational gradient and in response to experimental grazing, warming, and nitrogen addition treatments in Vestland County, Norway. Note that this dataset is split into a morphological and chemical trait data table; the latter will be populated on OSF as the data are ready. The chemical data set contains the additional variable ‘merged’ to indicate if several samples have been pooled to obtain enough biomass for the chemical analyses and the variable ‘ID_merged’ which lists the individual leaves in the merged sample, separated by underscore.

**Table 4:** Data dictionary for plant functional traits in the Warming Experiment (dataset i-b).

Variable name	Description	Variable type	Variable range or levels	Units	How measured
ID	Unique leaf ID	categorical	AAA7001 - HJZ2875		defined
date	Sampling date	date	2022-07-24 - 2022-07-30	yyyy-mm-dd	recorded
siteID	Unique site ID	categorical	Gudmedalen - Ulvehaugen		defined
elevation_m_asl	Elevation of site	numeric	1088 - 1213	m a.s.l.	recorded
blockID	Unique blockID	categorical	Gud_1 - Ulv_6		defined
warming	Warming treatment, C = control, W = warming	categorical	C - W		defined
individual_nr	Individual number	numeric	1–10		defined
species	Scientific name	categorical	*Achillea millefolium - Viola palustris*		identified
trait	Plant functional leaf trait	categorical	dry_mass_g - wet_mass_g		defined
value	Trait value	numeric	0 - 499.001	cm, g, cm^2^, mm, cm^2^ g^−1^, %, ‰	recorded
flowering	Individual was fertile	categorical	flower		recorded
comment	Comment on the data	categorical			recorded
problem	Describing the issue for flagging data	categorical			
flag	Flagging missing or unreliable data.	categorical	missing plotID - unreliable wet mass		recorded

Data and column descriptions for dataset i-b – plant functional traits from warmed and ambient climate plots from three sites along a precipitation gradient in Vestland County, Norway. Note that the functional trait dataset is split into a morphological and chemical trait dataset. The chemical data set contains the additional variable ‘merged’ to indicate if several samples have been pooled to obtain enough biomass for the chemical analyses and the variable ‘ID_merged’ which lists the individual leaves in the merged sample, separated by underscore.

**Fig. 4:**
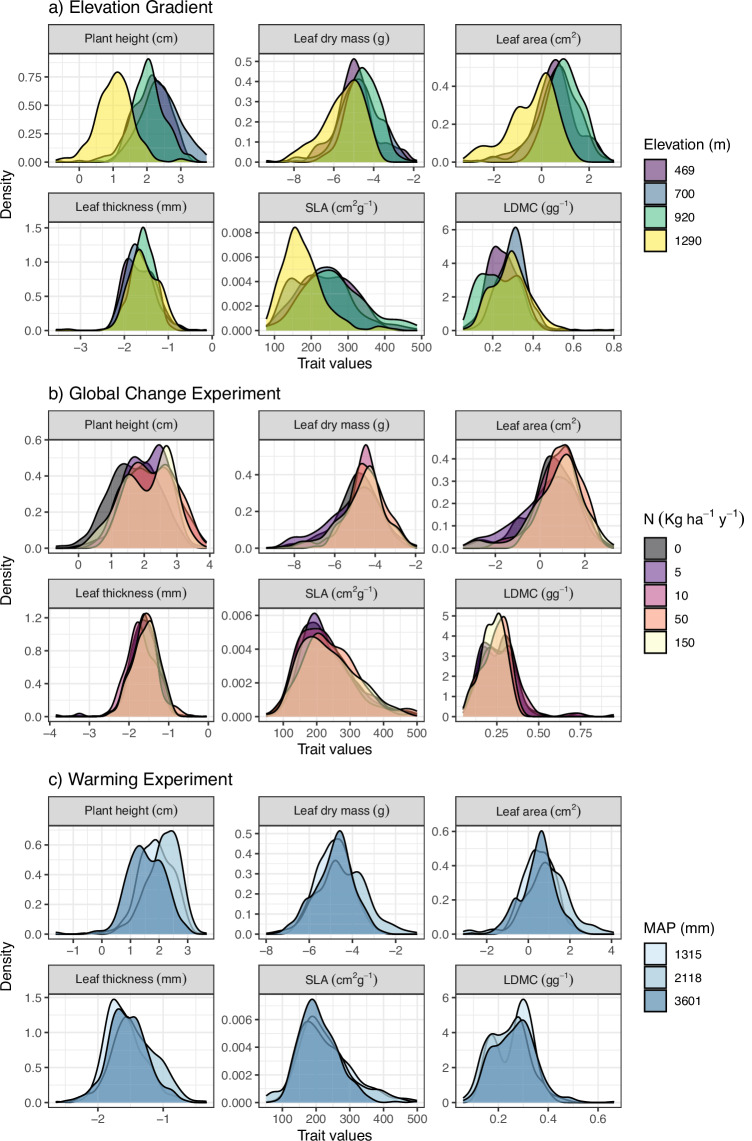
Plant functional trait distributions in response to elevation and global change drivers in mountain grassland vegetation in Vestland County, Norway. (**a**) Trait distributions across four sites along an elevational gradient (Elevation Gradient; dataset i-a). (**b**) Trait distributions in response to five Nitrogen addition treatments at the sub-alpine site in the Global Change experiment (dataset i-a). (**c**) Trait distributions across three sites differing in mean annual precipitation in the Warming Experiment (dataset i-b). Note that these are raw trait distributions (not community weighted) and are based on all sampled leaves for each site or treatment, so that grazing treatments are not separated on (**a**) and (**b**), and warming treatments are not separated on (**c**). Size traits (plant height, leaf area, leaf thickness and leaf dry mass) are log transformed. MAP = mean annual precipitation. Flagged data values denoting potentially erroneous LDMC values (n = 7) were removed prior to plotting.

Along the Elevation Gradient, morphological traits were measured for a total of 1,171 leaves and 54 taxa for a total of 8,086 unique trait observations (dataset i-a, Table 1, Fig. 4a). The number of morphological trait observations are relatively evenly distributed among sites and grazing treatments (770 [sub-alpine, ungrazed] – 1,312 [north boreal, grazed] unique measurements per grazing treatment and site). In the Global Change Experiment we measured morphological traits for 1,734 leaves from 55 taxa for a total of 11,949 unique trait observations, with on average 318 [range 70 – 494] unique trait measurements per plot (dataset i-a, Table 1, Fig. 4b). The total number of morphological trait measurements were higher in the plots without nitrogen addition because there were more replicate plots for this treatment. In the Warming Experiment, we measured morphological traits of 1,293 leaves from 38 taxa for a total of 8,795 unique trait observations, and chemical traits of 703 leaf samples from 39 taxa for a total of 3,661 unique trait observations (dataset i-b, Table 1, Fig. 4c). The number of morphological trait measurements and species sampled were lower in warmed plots [570 unique measurements, 31 species] than in ambient controls [723 unique measurements, 35 species].

### Dataset (ii): Leaf assimilation-temperature responses

Table 5 describes all variables in this dataset, which contains 122 raw and 88 clean assimilation-temperature curves for four species, *Agrostis capillaris*, *Alchemilla alpina, Achillea millefolium*, and *Vaccinium vitis-idaea* at the boreal, north boreal, sub-alpine, and alpine sites along the Elevation Gradient (Table 6).

**Table 5:** Data dictionary for leaf assimilation-temperature responses (dataset ii).

Variable name	Description	Variable type	Variable range or levels	Units	How measured
date_colleted	Date of turf collection	date	2022-07-23 - 2022-08-01	yyyy-mm-dd	recorded
date_measured	Date of AT measurement	date	2022-07-23 - 2022-08-01	yyyy-mm-dd	recorded
LICOR_name	Name of LI-COR LI-6800 machine used (refers to the lab that owns the machine)	categorical	1.Michaletz, 2.Michaletz, Pennell		recorded
curveID	Unique ID of AT curve	categorical	1000–1801		recorded
site_nr	Unique site number; 1 = Vikesland, 2 = Høgsete, 3 = Joasete and 4 = Liahovden.	numeric	1–4		defined
siteID	Unique site ID	categorical	Hogsete - Vikesland		defined
turf_number	Unique numeric ID of the turf in which leaf was located	numeric	1–10		recorded
elevation_masl	Elevation of site	numeric	469–1290	m	defined
species	Scientific name	categorical	*Alchemilla alpina* - *Vaccinium vitis-idaea*		identified

Data and column descriptions for dataset ii – leaf AT responses for four species from four sites along an elevational gradient and a warming, grazing, and nitrogen addition experiment in Vestland County, Norway. Note that default LI-6800 variables are not all explained in this data dictionary (for explanations see LI-6800 manual^103^). The factor levels in the LICOR.name column refer to the PI name of the labs owning the machines.

**Table 6:** Number of clean assimilation-temperature curves obtained for each species at each site along an elevational gradient in western Norway (Elevation Gradient).

Site (climate)	Vikesland (boreal)	Høgsete (north boreal)	Joasete (sub-alpine)	Liahovden (alpine)
*Agrostis capillaris*	0	2	0	0
*Alchemilla alpina*	8	10	8	7
*Achillea millefolium*	6	9	7	6
*Vaccinium vitis-idaea*	6	5	9	5

### Dataset (iii): leaf handheld hyperspectral readings

Table 7 describes all variables in this dataset, which contains leaf hyperspectral readings for 577 individuals from 12 species (Table 7) from the boreal (n = 232), north boreal (n = 112), sub-alpine (n = 167), and alpine sites (n = 70). The largest numbers of measurements were obtained from *Alchemilla alpina* (n = 154), *Vaccinium vitis-idaea* (n = 117), and *Achillea millefolium* (n = 83) (Table 8).

**Table 7:** Data dictionary for canopy leaf hyperspectral imagery (dataset iii).

Variable name	Description	Variable type	Variable range or levels	Units	How measured
species	Scientific name	character	*Achillea_millefolium – Veronica_alpina*		recorded
siteID	Unique site ID	character	Hogsete - Vikesland		recorded
turf_number	Unique numeric ID of the turf in which leaf was located	numeric	1–9		recorded
replicate	Replicate measurements per species per turf	numeric	1–12		recorded
‘338’ - ‘2515.2’	Proportion of light reflected by sample at each wavelength	numeric	0–1	Unitless	measured

Data and column descriptions for dataset iii – full range hyperspectral reflectance curves (338–2515 nm) from 12 species collected from turfs at four sites along an elevational gradient in Vestland County, Norway. Note that the dataset contains one column for each wavelength. For simplicity these have been merged to one row in the data dictionary.

**Table 8:** Number of hyperspectral readings taken for each species at each site (total measurements = 577).

Species	Vikesland (boreal)	Høgsete (north boreal)	Joasete (sub-alpine)	Liahovden (alpine)
*Achillea millefolium*	40	17	12	14
*Agrostis capillaris*	34	15	2	0
*Alchemilla alpina*	64	27	51	12
*Deschampsia flexuosa*	0	0	14	0
*Empetrum rubrum*	0	0	0	12
*Festuca rubra*	12	2	0	0
*Hypericum maculatum*	2	10	0	0
*Kindbergia praelonga*	48	0	0	0
*Rumex acetosa*	0	0	23	0
*Vaccinium myrtillus*	0	0	14	16
*Vaccinium vitis-idaea*	21	29	51	16
*Veronica alpina*	11	12	0	0

### Dataset (iv) canopy leaf temperatures

Table 9 describes all variables in this dataset, which contains diurnal time-series of thermal imagery on one day each at all sites along the Elevation Gradient. The alpine site was measured on 27^th^ of July 2022, sub-alpine on 28^th^ of July 2022, north boreal on 30^th^ of July 2022, and the boreal site on 1^st^ of August 2022. The time series data extend from approximately sunrise to sundown, except at the alpine site where data end at 18:36 due to a power source failure. In total, we obtained 2.26 billion raw temperature measurements. All timeseries include small gaps due to battery changes.

**Table 9:** Data dictionary for canopy leaf temperatures (dataset iv).

Variable name	Description	Variable type	Variable range or levels	Units	How measured
tc_soil1_c	Soil temperature	numeric	8.090704 - 17.85662	°C	recorded
tc_amb_c	Ambient air temperature	numeric	−0.7873993 - 19.22617	°C	recorded
tc_black_c	Reference plate temperature	numeric	0.01731279 - 49.18428	°C	recorded
ppfd_mV_raw	Photosynthetic photon flux density sensor raw signal	numeric	−0.03874302 - 7.596612	mV	recorded
ppfd_umol_m2_s	Photosynthetic photon flux density	numeric	−9.272754 - 1818.173	μmol m^−2^ s^−1^	recorded
Date	Date and time	date	220727-054314 - 220731-132334	yymmdd-hhmmss	recorded

Data and column descriptions for dataset iv - canopy leaf temperatures from four sites along the Elevation Gradient in Vestland County, Norway. Main response variables only are listed in this table; additional variables related to thermal camera calibration are included in the data files. Note that this dataset is split into different datasets stored in different folders, one per site.

### Dataset (v): ecosystem CO_2_ fluxes

Table 10 describes all variables in this dataset, which contains the diurnal ecosystem CO_2_ fluxes, measured hourly for 24 h at each of the four sites along the Elevation Gradient and in the warmed and ambient climate treatments of the Global Change Experiment. Each measurement has a paired light (NEE) and dark (ER) observation, which were used to calculate GPP (418 observations of each flux type). The dataset also provides soil and air temperature and PAR values recorded during flux measurements. See the folder R_code/data_cleaning in the GitHub repository^84^ for code to download and clean the data.

**Table 10:** Data dictionary for ecosystem CO_2_ fluxes (dataset v).

Variable name	Description	Variable type	Variable range or levels	Unit	How measured
datetime	Date and time of the carbon flux measurement	date_time	2022-07-23 21:45:15 - 2022-07-31 08:12:45	yyyy-mm-dd hh:mm:ss	recorded
time	Time of the carbon flux measurement (independent of date)	date_time	00:00:43 – 23:59:32	hh:mm:ss	recorded
origSiteID	Unique site ID of origin site	categorical	Hogsete - Vikesland		defined
destSiteID	Unique site ID of destination site (the site they were measured at)	categorical	Hogsete - Vikesland		defined
turfID	Unique turf ID as origPlotID, treatments, destPlotID	categorical	105 WN3C 173 - TTC 146		defined
warming	Warming treatment with W for warming or A for ambient	categorical	A - W		defined
type	Types of CO2 flux data: GPP = Gross Primary Productivity, NEE = Net ecosystem exchange, ER = ecosystem respiration	categorical	ER - NEE		defined
fluxID	Unique identifier for each flux measurement	numeric	1 - 288		defined
flux_value	Flux slope. Corrected for CO2 accumulation in canopy at night.	numeric	−141.365 - 130.504	mmol /m^−2^ /hr^−1^	calculated
PARavg	Mean Photosynthetic Active Radiation (PAR)	numeric	−2.843 - 1840.27	µmols^−1^ sqm^−1^	recorded
temp_soil	Mean soil temperature measured during flux measurements.	numeric	0.84 - 32.13	°C	recorded
temp_airavg	Mean air temperature measured during flux measurements.	numeric	0.807 - 32.454	°C	recorded
flag	Flagging missing or unreliable data.	categorical	discard - zeroNEE		defined

Data and column descriptions for dataset v – ecosystem CO_2_ fluxes at four sites along an elevational gradient and a warming and grazing experiment in Vestland County, Norway (Global Change Experiment and Elevation Gradient).

### Dataset (vi-a and vi-b): landscape-scale airborne multispectral imagery

Table 11 describes all variables and technical specifications for the landscape-scale airborne multispectral imagery (see Fig. 3). The clean orthomosaics can be found on the OSF repository^68^.

**Table 11:** Summary of dataset vi remote sensing data generated for this paper.

(A) Site name	RGB flights (DJI Mavic 2 Pro)						Spectroscopy/Trait collection	
	Area (ha)	Pixel Resolution (cm)	Latitude (°N)	Longitude (°E)	dGPS GCPs	Number of geolocated images (×10 bands)	Single-species turfs for spectroscopy & traits (spectra read)	Number Species measured through field spectroscopy
Boreal	4.98	2.00	608.811	7.1659	9	369	21 (232)	8
North boreal (full site)	4.61	1.45	608.770	7.1717	11	873	16 (112)	7
Sub-alpine	—	—	—	—	—	—	9 (167)	7
Alpine	—	—	—	—	—	—	7 (70)	5
TOTAL/AVERAGE	9.59	*1.73*	*608.791*	*7.1688*	*10*	1,242	53 (581)	12
(B) Site name	UAV 10-band flights (DJI Inspire 2 w/ MicaSense Dual Camera)							
	Area (ha)	Pixel Resolution (cm)	Latitude (°N)	Longitude (°E)	dGPS GCPs	Number of geolocated images (×10 bands)	Georeferenced extra ground points	
							GNSS vegetation ground truthing points	Number of species/functional types in ground truthing points
Boreal	4.98	5.71	608.818	7.1655	10	161	31	14
North boreal (full site)	4.61	4.11	608.773	7.1715	11	1,927	78	19
Sub-alpine	0.71	2.06	608.626	7.1668	10	681	59	16
Alpine	0.38	2.57	608.607	7.1937	7	278	39	14
TOTAL/AVERAGE	10.69	*3.62*	*608.706*	*7.1744*	*9.5*	3,047	207	40
(C) Site name	RGB flights (DJI Mavic 2 Pro) - sfm flight							
	Area (ha)	Pixel Resolution (cm)	Latitude (°N)	Longitude (°E)	dGPS GCPs	Number of geolocated images (×10 bands)	Point Cloud (Number of points)	RMS Reprojection Error (pix)
North boreal - sfm	0.01	0.10	608.755	7.1760	5	224	55,497,905	2.956

(a) RGB flights: main characteristics of the orthomosaics built for study sites, which consists of a true colour (RGB) TIFF file. (b) UAV 10-band flights: main characteristics of the orthomosaics built for each study site. For each flight, radiometrically calibrated reflectance values exist for 10 bands: Coastal Aerosol (444 ± 28 nm), Blue (475 ± 20 nm), Green (531 ± 14 nm and 560 ± 20 nm), Red (650 ± 16 nm and 668 ± 10 nm), Red Edge (705 ± 10 nm, 717 ± 10 nm, and 740 ± 15 nm), and Near Infrared (840 ± 40 nm). (c) For the north boreal Høgsete site - sfm, a dense 3D point cloud was generated. Latitude and Longitude correspond to the origin of the raster (north-west corner); Number of geolocated images shows the number of individual overlapping images used to build each orthomosaic. Spectroscopy/Trait collection: information on the single-species turfs collected for ground-truthing, leaf spectroscopy (dataset **iii**), and trait measurements (dataset i). Georeferenced extra ground points: number and species (or functional group) of identified vegetation types in the field which were georeferenced with a differential GNSS system.

#### Dataset vi-a orthomosaics

Table 11a describes all variables and technical specifications in the very high resolution RGB (truecolour) reference orthomosaics, which were created for the boreal and north boreal sites at spatial resolutions of 2 cm and 1.45 cm, respectively (Fig. 3c). Table 11b describes all variables and technical specifications in the multispectral (10-band) imagery produced for each site and for each of the 10 reflectance bands: Coastal Aerosol [444 ± 28 nm], Blue [475 ± 20 nm], Green [531 ± 14 nm and 560 ± 20 nm], Red [650 ± 16 nm and 668 ± 10 nm], Red Edge [705 ± 10 nm, 717 ± 10 nm, and 740 ± 15 nm], and Near Infrared [840 ± 40 nm]). The spatial resolutions of these orthomosaics ranged from 2.06 cm to 5.71 cm depending on the site (Table 11b). A variety of vegetation indices can be readily obtained combining some of these reflectance bands, such as NDVI^85^ and EVI^86^ (Fig. 3d).

#### Dataset vi-b photogrammetry

Table 11c describes the photogrammetry exercise done over an area dominated by juniper shrubs with an herbivore exclosure resulted in a dense point cloud of >55 M points over an area of 0.01 ha (Fig. 3b). A reference orthomosaic was also built with a pixel resolution of 0.01 cm.

### Dataset (vii): microclimate

Table 12 describes all variables in the clean plot-level near-surface ground and soil temperature and soil moisture data, measured with TOMST TMS-4 dataloggers in 15 min intervals at the same plots as the CO_2_ flux measurements (dataset v) during the 2022 field campaign. We measured near-surface temperature 2,554 times, ground temperature 2,554 times, soil temperature 2,549 times, and soil moisture 2,531 times, for a total of 10,188 observations. For more extensive climate data see the Vestland Climate Grid, ThreeD, and INCLINE project data papers and repositories^63–65^. The code to download and clean the data is provided in the folder R_code/data_cleaning in the GitHub repository^84^.

**Table 12:** Data dictionary for microclimate data (dataset vii).

Variable name	Description	Variable type	Variable range or levels	Unit	How measured
datetime	Date and time of measurement	date_time	2022-07-23 00:15:00 - 2022-08-08	yyyy-mm-dd hh:mm:ss	recorded
loggerID	Unique climate logger ID	categorical	95221106 - 94201707		defined
turfID	Unique ID of vegetation turf as origPlotID, treatments and destPlotID	categorical	100 AN5M 100 - TTC 146		defined
origSiteID	Unique origin site ID	categorical	Hogsete - Vikesland		defined
destSiteID	Unique destination site ID	categorical	Hogsete - Vikesland		defined
warming	Warming treatment with W for warming or A for ambient	categorical	A - W		defined
datetime_in	Date and time the logger was installed or date and time the logger data should be trimmed to	date_time	2019-08-22 23:00:00 - 2022-07-29 22:30:00	yyyy-mm-dd hh:mm:ss	defined
datetime_out	Date and time the logger was removed or date and time the logger data should be trimmed to	date_time	2022-07-28 11:00:00 - 2022-09-02 06:00:00	yyyy-mm-dd hh:mm:ss	defined
climate_variable	Microclimate variable	categorical	air_temperature - soil_temperature		defined
value	Temperature or moisture reading including values later flagged as suspect	numeric	−1 - 34.625	°C, (m3 water × m^−3^ soil) × 100	recorded

Data and column descriptions for dataset vii - microclimate at four sites along an elevational gradient and a warming and grazing experiment in Vestland County, Norway (Global Change Experiment and Elevation Gradient).

## Technical Validation

### Experimental validation

Further information on the experiments and experimental validation can be found in^26–29,87^.

### Taxonomic validation

A large number of people were involved in plant functional traits data collection, which introduces a risk of observer errors, particularly misidentification of difficult taxonomic groups and sterile or grazed specimens. To detect and correct such errors in the trait data, the experts working in this region (AHH, VV) checked all specimens during data collection in the field or before processing in the lab. The taxonomy was checked and corrected against the Taxonomic Name Resolution Service (TNRS)^88^. A list of all identified species across datasets is also available in the taxon table in the OSF repository (see Fig. 2). The dataset contains one *Carex* specimen identified only to the genus level (*Carex* sp.).

### Trait data validation

Trait data were thoroughly checked, validated, corrected and flagged as follows. First, duplicate observations were removed. We then checked and, if possible, corrected missing or erroneous sample or treatment identifications against field notes and notes on the envelope labels. In a few cases this was not possible (missing blockID: n = 1, and missing grazing treatment: n = 2). Third, unrealistically high or low trait values were checked and corrected against the lab and field notes for typing errors and corrected as appropriate. We then checked the data by visualisation (e.g., plotting leaf wet mass vs. dry mass), identified any outliers, and corrected errors if possible. Scans for leaf area were manually corrected where possible by editing out non-leaf objects (n = 28), filling in ‘white’ leaf area (n = 19) or adjusting cropping (n = 19). Leaf area was recalculated after these edits. To adjust cropping, settings for recalculating the leaf area were changed, for example to crop the scans differently to remove black lines at the edges. If corrections were not possible, samples with clearly unrealistic trait values were flagged to be removed from the dataset (see the code for details on each specific case). This was done for leaves with dry matter values higher than 1 g g^−1^, specific leaf area values smaller than 5 cm^2 ^g^−1^ or greater than 500 cm^2 ^g^−1^ (n = 53 total values removed), and leaf nitrogen values higher than 6.4%. The nitrogen cutoff values were chosen based on the highest published leaf nitrogen values found in the Botanical Information and Ecology Network^89,90^ for the genera in our study. Finally, not all the issues in the data could be resolved, including those arising from partially damaged leaves, missing petiole or stipules, folded or overlapping leaflets on scans, measurements made on wilted leaves, or recorder errors, which leads to (potential) issues with specific trait datapoints (e.g., SLA if there is a potential error in leaf area or dry weight). We added two columns to the trait datasets to help the user make decisions for how to handle such data depending on their needs and priorities. The column “problem” describes what the problem in the data is (e.g., overlapping leaves) and the column “flag” describes the effect this has on specific trait measurements (e.g., area < expected). We left all comments from the data processing in the dataset that might be useful for the users (e.g., notes on corrections on leaf scans, recalculation of dry mass, when leaves were lost). The data checking code and outcomes for these various procedures is available and documented in the code and associated readme file. All changes and corrections are noted in the comments column for individual samples.

### Leaf assimilation-temperature response validation

Some of the measured AT curves were unfit for the purposes of the study, so we used objective exclusion criteria to eliminate unsatisfactory curves as follows. We fit cubic smoothing splines to each curve and eliminated any curves in which the fitted splines exhibited multimodality (i.e., more than one maximum point). For each curve, we additionally checked the fitted optimal temperature (*T*_*opt*_) value from the Sharpe-Schoolfield model with high temperature deactivation^91^, reformulated with an explicit *T*_*opt*_ parameter^92^, and if the fitted *T*_*opt*_ value was less than the lowest measured leaf temperature or greater than the highest measured leaf temperature, we excluded the curve (total of n = 61 exclusions). This ensures our AT curves adequately captured the *T*_*opt*_ region while excluding curves with confounding issues such as stomatal oscillation^93^.

### Canopy leaf temperatures validation

To produce time-series of mean canopy temperatures at each site, thermal images were analysed using MATLAB code^76^ modified for use with our apparatus (https://github.com/MichaletzLab/thermal_analysis_pftc6).

### Ecosystem CO_2_ flux validation

While cleaning the data, we found that our results were altered by the CO_2_ that had accumulated in the vegetation layer before the NEE measurements. This layer was disturbed before the ER measurements, leading to erroneous GPP measurements that were particularly noticeable during the night when no photosynthesis was expected to occur (see^94,95^). To correct for this effect, we adjusted each 24 h cycle of fluxes at each site so that the most positive GPP measurement equalled zero. Users who wish to use uncorrected data are referred to the final lines in the GitHub code^84^ for adjusting the processing each individual site.

Fluxes starting at a concentration outside of 421 ± 100 ppm were flagged and removed from the clean data (n = 136). The quality of the fit was assessed by setting a threshold on the b parameter. Because the b parameter is inside the logarithmic term, fluxes for which b >  = 1 were discarded. The parameter b sometimes fell out of the range [0:1] as an artefact of how the base::optim function finds local minimums for C(t). We also considered ER fluxes with a negative slope (uptaking CO_2_) as bad fits. If the fit was determined as bad (based on one of those thresholds) but there was a correlation (|coefficient| > 0.5) between time and CO_2_ concentration, the flux was discarded (n = 10). However, if the fit was bad and there was no correlation between CO_2_ concentration and time, the flux was replaced by 0 as the variation is mostly explained by noise in the system (n = 6). After visual inspection, we flagged and removed from the clean data 18 out of 1,296 flux measurements. All fluxes were labelled with a flag indicating quality (Table 13). If GPP was calculated from fluxes that were replaced by 0, it has been flagged with zeroNEE or zeroER. Missing rounds of measurements are indicated as such. microclimate data.

**Table 13:** Explanation of the quality flag column in dataset v - ecosystem CO_2_ fluxes.

flag	Replaced flux value	explanation	Number of fluxes (including GPP)
ok	flux	—	1094
start_error	NA	The flux is starting at a value that is out of the range 421 ± 100 ppm	136
discard	NA	Bad fit with a correlation between time and CO_2_ concentration	10
weird_flux	NA	The changes in CO_2_ concentration over time do not have a reasonable natural explanation	17
zero	0	Bad fit and no correlation between time and CO_2_ concentration	6
zeroER (GPP only)	GPP calculated with ER = 0	ER was flagged as “zero”	5
zeroNEE (GPP only)	GPP calculated with NEE = 0	NEE was flagged as “zero”	1
Missing round	NA	Rounds of measurement that were missed on the field	54

**Table 14:** Explanation of the quality flag column in dataset vii-microclimate.

flag	Replaced value	explanation	Number of measurements
cut_min_moist	NA	Moisture is below reasonable minimum (0%)	47
cut_Tmax_air	NA	Air temperature is above reasonable maximum (30 °C)	46
cut_Tmax_ground	NA	Ground temperature is above reasonable maximum (35 °C)	2
cut_Tmax_soil	NA	Soil temperature is above reasonable maximum (20 °C)	74
cut_Tmin_soil	NA	Soil temperature is below reasonable minimum (5 °C)	38

Some ecosystem carbon flux measurements are missing due to logistical issues, or equipment failure (specifically, the alpine site at 00:00, and the sub-alpine site between 18:00 and 19:30). Another equipment failure at 03:00 at the sub-alpine site required us to resume measuring 24 hours later.

### Microclimate validation

We removed outlier microclimate data when the temperatures were beyond reasonable expected minimum or maximum temperatures (−40 °C to 30 °C for air temperature, −40 °C to 35 °C for ground temperature, 5 °C to 20 °C for soil temperature; n = 5) or unreasonably low soil moisture (<0%) (Table 14). Some loggers used had been installed by the ThreeD project. Those data were downloaded and combined with our dataset. For consistency, the same filters used on the PFTC6 data were applied to the ThreeD data. These quality thresholds removed 154 data points (all sensors together) out of 454,656 entries.

## Usage Notes

### Data use and best practice

The data are provided under a CC-BY licence. We suggest that data presented here and accessed through the OSF, including future additions to the chemical trait data, be cited to this data paper. We appreciate being contacted for advice or collaboration, if relevant, by users of these data. In cases where our data make up >10% of the data used in downstream publications we anticipate that appropriately acknowledging our contributions would result in an invitation for collaboration.

### Relation to other datasets

The data presented here relates to a large amount of site-, block-, and plot-level data from sites and experiments from within the Vestland Climate Grid^26–28,96,97^, ThreeD, and INCLINE^29^ projects, the most relevant of which are briefly explained under ‘Background and other datasets’ above. These data are available in the Vestland Climate Grid^64^, ThreeD^63^, and INCLINE^29^ projects and OSF repositories, and can be linked to the data described here through various keys, including species and plots (see Fig. 2), allowing the combination of data from this paper with e.g., vegetation composition for community-weighted trait distribution analyses. Examples of code to access and download relevant datasets from these repositories is provided in the code^66^ (*other_code/download_comm_data.R*). See Fig. 2 for a conceptual representation of how these datasets are linked via shared variables/keys.

### Data quality

The procedures for and consequences of various decisions during data collection, management, and cleaning are detailed in this paper, and in the associated code^66,82–84^. The code describes and implements our suggested data cleaning and checking procedures that result in producing what we consider the clean and ‘best practice’ final datasets. The various ‘flag’, Tables 3–12) indicate reasons why specific datasets were removed, and/or can be used to identify additional specific data points that could be removed to create even more robust datasets. Further details on the flag columns in the carbon flux and microclimate datasets are given in Tables 13, 14. Users who might prefer stricter or more inclusive data handling strategies should check the flags in the clean and raw data sets and adjust the data cleaning accordingly.

### Data naming and combination

The plant functional trait datasets are split into separate files for the morphological and chemical traits, which again are split into one file for the Elevation Gradient/Global Change Experiment and one for the Warming Experiment. We split the different experiments because the research projects on which these studies are based have different treatments and naming conventions and we think they will rarely be combined. The morphological and chemical traits were split because the chemical traits contain pooled samples. The morphological and chemical datasets are compatible and can be combined by for example using the *bind_rows()* function in R^98^ or similar. Note that the chemical trait datasets contain duplicate observations for pooled samples to link the information to all individuals in a pooled sample (indicated in column *merge*). Users should be aware and could remove these duplicates from analyses when relevant. Note also that all the chemical trait datasets are not yet complete at the time of publication but will be updated on OSF as the data are ready.

### Other notes and resources

The correct settings for the leaf area scanning was automatically checked by using a raspberry pi set up connected to the scanner. The setup was developed by the PFTC core team and is documented on the GitHub repository^99^.

The flux fitting method is currently being turned into an R package with all calculations and the steps described here integrated as functions^100^.

## Data Availability

The code used for checking, cleaning and analysing the data is available in the following GitHuB repositories^66,83,84^. Note that these references refer to versioned copies of the repositories available on Zenodo, live versions can be found at GitHub.
